# A novel aromatic oil compound inhibits microbial overgrowth on feet: a case study

**DOI:** 10.1186/1550-2783-4-3

**Published:** 2007-07-13

**Authors:** Bill D Misner

**Affiliations:** 1 West 1140 Glass Avenue Spokane, Washington, 99205, USA

## Abstract

**Background:**

Athlete's Foot (Tinea pedis) is a form of ringworm associated with highly contagious yeast-fungi colonies, although they look like bacteria. Foot bacteria overgrowth produces a harmless pungent odor, however, uncontrolled proliferation of yeast-fungi produces small vesicles, fissures, scaling, and maceration with eroded areas between the toes and the plantar surface of the foot, resulting in intense itching, blisters, and cracking. Painful microbial foot infection may prevent athletic participation. Keeping the feet clean and dry with the toenails trimmed reduces the incidence of skin disease of the feet. Wearing sandals in locker and shower rooms prevents intimate contact with the infecting organisms and alleviates most foot-sensitive infections. Enclosing feet in socks and shoes generates a moisture-rich environment that stimulates overgrowth of pungent both aerobic bacteria and infectious yeast-fungi. Suppression of microbial growth may be accomplished by exposing the feet to air to enhance evaporation to reduce moistures' growth-stimulating effect and is often neglected. There is an association between yeast-fungi overgrowths and disabling foot infections. Potent agents virtually exterminate some microbial growth, but the inevitable presence of infection under the nails predicts future infection. Topical antibiotics present a potent approach with the ideal agent being one that removes moisture producing antibacterial-antifungal activity. Severe infection may require costly prescription drugs, salves, and repeated treatment.

**Methods:**

A 63-y female volunteered to enclose feet in shoes and socks for 48 hours. Aerobic bacteria and yeast-fungi counts were determined by swab sample incubation technique (1) after 48-hours feet enclosure, (2) after washing feet, and (3) after 8-hours socks-shoes exposure to a aromatic oil powder-compound consisting of *arrowroot, baking soda, basil oil,  tea tree oil, sage oil, and clove oil*.

**Conclusion:**

Application of this novel compound to the external surfaces of feet completely inhibited both aerobic bacteria and yeast-fungi-mold proliferation for 8-hours in spite of being in an enclosed environment compatible to microbial proliferation. Whether topical application of this compound prevents microbial infections in larger populations is not known. This calls for more research collected from subjects exposed to elements that may increase the risk of microbial-induced foot diseases.

## Background

Kobayashi [12] isolated a gram-positive cocci bacterium from foot skin of 17 volunteers by swab sampling technique. This bacterium was identified as Staphylococcus epidermidis. Except for this unique staphylococcal species, microbes isolated from the athlete's foot infections are typically colonies of yeast-fungi. There are 700 known classified yeast-fungi, although their colonies often look like bacteria. Uncontrolled proliferation of yeast-fungi produces small vesicles, fissures, scaling, and maceration with eroded areas between the toes and the plantar surface of the foot, resulting in intense itching, blisters, and cracking. Athlete's Foot (Tinea pedis) is a form of ringworm associated with highly contagious colonies of Epidermophyton floccosum, Microsporum canis, Trichophyton mentagrophytes and Trichophyton rubrum.

Painful microbial foot infection may prevent athletic participation. Keeping the feet clean and dry with the toenails trimmed reduces the incidence of skin disease of the feet. Wearing sandals in locker and shower rooms prevents intimate contact with the infecting organisms and alleviates most foot-sensitive infections. Hot weather, sweating, exercise, and shoes, generates a moisture-rich environment that stimulates overgrowth of both aerobic bacteria and yeast/fungi. The most harmful interdigital infections begin with invasion of the horny layer by dermatophytes. Large numbers of normally resident diphtheroids cause the common wet, macerated Athlete's Foot Syndrome *(Tinea pedis)*, due to an overgrowth of gram-negative organisms. The dry, scaly type (dermatophytosis simplex) alternates with the wet, macerated type (dermatophytosis complex). Flare-ups are more common in summer and are experimentally induced by occlusion of fungus-infected feet. The pustular-mid sole form responds best to topical antifungal agents, while the interdigital form, responds to a mixed treatment of anti-fungal/anti-bacterial antibiotics, regular drying routine, and constant debridement. Toenail infections are caused by a variety of organisms, which may appear as onycholysis with or without paronychia and must also be treated with appropriate antibiotics. Since these microbes are part of the normal flora, infection often reoccurs, necessitating regular treatment [8]. Suppression of microbial growth is accomplished by exposing the feet to air (e.g., wearing sandals) to enhance evaporation to reduce moistures' growth-stimulating effect. Drying is the decisive element and is often neglected. Topical antibiotics present a potent approach with the ideal agent being one that dries the skin by absorbing moisture and generating broad-spectrum antibacterial-antifungal activity. Potent agents virtually exterminate interdigital dermatophytes, but the inevitable presence of infection under the nails predicts future infection [14,20]. There is an association between microbial overgrowth and disabling foot infections in athletes. Common skin microbes proliferate rapidly in a moisture-rich, enclosed environment resulting in limited or no athletic participation. Resolution of a severe infection may require prescription drugs, salves, or foot soakings. *Arrowroot, baking soda, basil oil,  tea tree oil, sage oil, and clove oil *are also reported to inhibit both aerobic bacteria and yeast-mold-fungi growths respectively [1-7,9-11,13,15-19,21-23].

## Methods

An aromatic oil powdered compound was selected for its broad-spectrum antibiotic effects on the feet of a single subject. The compound was selected to measure its combined antibiotic effect inhibiting aerobic bacteria or fungi/yeast cultures growing on the confined surfaces of the feet. A female volunteer stimulated the growth of common microbes by wearing socks and shoes for 48-hours without bathing or exposure to dry air. A significant increase in aerobic bacteria and yeast-fungi-mold concentrations were detected in the first incubated sample set. Application of the antibacterial-antifungal aromatic oil compound to feet reduced aerobic bacteria by a factor of 10,000 (from 100,000,000 to 10,000 per milliliter), and retained this suppressed number for 8 consecutive hours in spite of the subject's return to wearing socks and shoes. Furthermore, application of the antibacterial-antifungal aromatic oil compound to the feet surprisingly inhibited the yeast-fungi count by a factor of 100 (from 100,000 to 1000 per milliliter), and retained this suppressed number for 8 consecutive hours in spite of the subject's return to wearing socks and shoes. The application of this compound was associated with a remarkable inhibition of time/moisture-induced microbial overgrowth in this subject's feet.

Biosan Laboratories microbial test kits were utilized to sample and determine aerobic bacteria (AB) and yeast-fungi-mold (YFM) counts by an incubation procedure. A single female subject (63 y) enclosed feet in socks and shoes without bathing, without exposure to air for 48-hours. Three sets of 2 swab samples were extracted between-toes and subsequently incubated @ 25°–30°C (77–86°F) to determine the numerical count of aerobic bacteria and yeast-fungi/mold in the following samples:

1. Aerobic Bacteria & Yeast-Fungi count enclosed environment 48-hours no bathing

2. Aerobic Bacteria & Yeast-Fungi count immediately after washing with soapy water, drying, and application of *arrowroot, baking soda, basil oil, bay oil, tea tree oil, sage oil, and clove oil *powder

3. Aerobic Bacteria & Yeast-Fungi count 8-hours wearing socks and shoes following application of *arrowroot, baking soda, basil oil, bay oil, tea tree oil, sage oil, and clove oil *powder return to for

## Results

**Figure 1:**
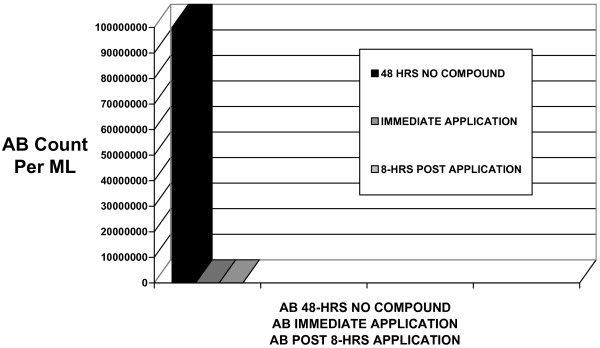
Aerobic Bacteria (AB) Inhibited By Powder Compound Application.

**Figure 2:**
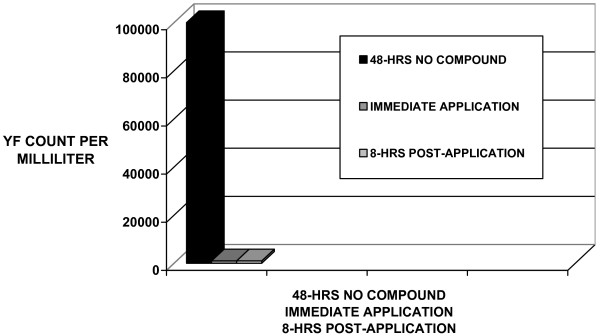
Yeast-Fungi Inhibited By Compound Application.

The sample extracted from feet enclosed in socks and shoes for 48-hours yielded a 100,000,000 aerobic bacteria count and a 100,000 yeast-fungi-mold count. After application of *arrowroot, baking soda, basil oil, bay oil, tea tree oil, sage oil, and clove oil *powder compound, the aerobic bacteria count was remarkably reduced 10,000 times less, from 100,000,000 to 10,000 aerobic bacteria per milliliter swab sample. After application of *arrowroot, baking soda, basil oil, bay oil, tea tree oil, sage oil, and clove oil *powder compound, the yeast-fungi count were remarkably reduced 100 times, from 100,000 to 1000 per milliliter swab sample (See Table 1 and Figures 1 &2, AB & YFM Inhibition Associated with application of a natural Aromatic Oil Compound).

**Table 1:** AB & YF count before and after powder application

Test	AEROBIC BACTERIA Per Milliliter	YEAST-FUNGI Per Milliliter
Pre-Test 48-Hours Enclosed (in socks & shoes) No air, No Bathing	>100,000,000	>100,000
Immediately After Bathing & Powder Compound Application	>10,000	>1000
Eight-Hours After Powder Compound Application Feet Enclosed (in socks & shoes)	>10,000	<1000

## Conclusion

Application of this novel herbal powdered compound to the external surfaces of feet inhibited overgrowth of aerobic bacteria and yeast-fungi-mold immediately, and continued to limit microbial proliferation for 8-hours. Whether topical application of this compound prevents microbial infections in larger populations is not known. This calls for more research collected from subjects exposed to elements that may increase the risk of microbial-induced foot diseases.

## Competing interests

All work has been completed in accordance with guidelines governing such work with no financial relationships (including grants, honorarium, stipends, patents or patents pending, royalty agreements, board memberships, and/or consultant relationships) related to these findings and has no remunerative nor competing interests from the natural compound selected for this project.
